# Farming systems in sheep rearing: Impact on growth and reproductive performance, nutrient digestibility, disease incidence and heat stress indices

**DOI:** 10.1371/journal.pone.0244922

**Published:** 2021-01-13

**Authors:** D. Karthik, J. Suresh, Y. Ravindra Reddy, G. R. K. Sharma, J. V. Ramana, G. Gangaraju, Y. Pradeep Kumar Reddy, D. Yasaswini, M. J. Adegbeye, P. Ravi Kanth Reddy

**Affiliations:** 1 Department of Livestock Production Management, College of Veterinary Science, Sri Venkateswara Veterinary University, Tirupati, India; 2 Center for Continuing Veterinary Education & Communication, College of Veterinary Science, Sri Venkateswara Veterinary University, Tirupati, India; 3 Department of Veterinary and Animal Husbandry Extension Education, College of Veterinary Science, Sri Venkateswara Veterinary University, Tirupati, India; 4 Controller of Examination, College of Veterinary Science, Sri Venkateswara Veterinary University, Tirupati, India; 5 Livestock Research Station, Sri Venkateswara Veterinary University, Palamaner, India; 6 Department of Veterinary Medicine, College of Veterinary Science, Sri Venkateswara Veterinary University, Tirupati, India; 7 Department of Animal Science and Livestock Production, College of Agriculture and Natural Sciences, Joseph Ayo Babalola University, Ikeji-Arakeji, Ilesha, Osun State, Nigeria; 8 Veterinary Assistant Surgeon, Animal Husbandry Department, Veterinary Dispensary, Taticherla, Prakasam District, Andhra Pradesh, India; Tokat Gaziosmanpasa University, TURKEY

## Abstract

The experiment was conducted with an intent to know the effect of different farming systems on the growth performance, nutrient digestibility coefficients, reproductive traits, disease incidence, heat stress indices, and cost economics of Nellore sheep. The study includes two parallel trials to prevent the influence of age on heat stress indices (panting score and erythrocyte osmotic fragility (EOF)). One hundred and twenty lambs (60 ram-lambs and 60 ewe-lambs) were allotted in a randomized block design under extensive, semi-intensive, and intensive systems for trial I, whereas trial II include eighteen rams assigned to the three respective farming systems in a completely randomised design. Both, season (summer) and grazing practice increased the panting score and EOF. The heat stress indices were positively correlated (P<0.01) with dry-bulb temperature and temperature-humidity index (THI) and inversely correlated (P<0.01) to relative humidity. Allotting the sheep to intensive system increased (P<0.001) weight gain and average daily gain with higher effect in males compared to females. The parameters of asymptotic weight (A), integration constant (B), and maturation rate were higher for intensive males. The male Nellore lambs had higher asymptotic weight and lower maturity rate than females, irrespective of the rearing system. Intensive sheep revealed a higher dry matter intake, digestibility coefficients, feed conversion ratio. The instantaneous bite mass (IBM) was higher for *Commelina benghalensis*, while instantaneous bite frequency (IBF), instantaneous intake rate (IIR) were higher for *Cyanodon dactylon* and *amaranthus viridis*, respectively. The proportion of intakes were highest for *Stylo hemata* followed by *Cynodon dactylon* and *Tridax procumbens* species. No differences were observed for the weight at puberty, oestrus cycle length, oestrus duration, conception percent, gestation period, and lambing percent in three rearing systems; however, the age at puberty was lower (P<0.001) and the birth weight was higher (P<0.001) for sheep reared under intensive farming system. Highest disease incidence was observed in rainy and winter seasons, particularly in sheep reared under extensive system. The capital expenditure was same for the three rearing systems, while the recurring expenditure was higher for Intensive farming system. The gross income and net income were higher for intensive system on account of higher weight gains. However, the higher returns per rupee of expenditure project the extensive farming as an ideal rearing system for small farmers and entrepreneurs with a low initial capital.

## 1. Introduction

Most of the developing countries of the world are found in the tropics, which are currently experiencing a rapid hike in human population, dramatic urbanization, monetarization of economics, and income change. Thus, the major issues to be addressed for these countries include enhancement of food security by combating poverty and achieving agricultural growth that would contribute to overall economic development [1]. Sheep with multi-facet utility (for meat, wool, skin, manure and to some extent milk) plays a vital role in the Indian agrarian economy. They are better adapted to arid and semi-arid tropics with marginal and sub-marginal lands. They are perhaps the most suitable small ruminants to utilize the sparse vegetation available in dryland areas through rangeland management and reseeded pastures [2].

Sheep in India are mostly maintained on natural vegetation, common grazing lands, wastelands, uncultivated (fallow) lands, stubbles of cultivated crops, and top feeds (tree toppings). Few farmers rear the sheep even on grain, cultivated fodder, and crop residue. In developing countries such as India, the farming system could be categorized as intensive, semi-intensive, and extensive systems. In intensive farming, the animals are fed in confinement with no access to graze. The system involves high cash inputs. In extensive and semi-intensive farming system, the sheep flocks are let loose for a grazing period of 4–8 hours. This practice helps in increasing the fertility of land via the return of dung and urine, control of waste herbage growth, reduced fertilizer usage, easier crop management, increased crop yields, and greater economic returns. Extensive system or pastoralism involves low carrying capacity in situations where land is marginal and plentiful, which is characterized by low rainfall and grazing. Extensive farming is a way of life in several geographies, including Australia, Africa, India, Eurasian steppes, Tibetan plateau, and many third world countries [3]. Most of the time, no well-defined pasturelands are available for sheep, and they mainly depend on wastelands, which are otherwise not suitable for crop production.

Another concern for the semi-intensive and extensive farming systems is the compromised animal welfare because of heat stress. Developing practical technologies that assist in detecting welfare issues are necessary [4]. Body temperature is the most reliable indicator of heat stress; however, its determination under field conditions is arduous [5]. Hence, developing least invasive methods to quantify the heat load is of great importance. Panting scores, a proxy of body temperature, could be easily recorded without disturbing the animals. Erythrocyte osmotic fragility (EOF) tests is another simple, yet effective method in measuring both heat load status and thermal resilience of sheep [6].

Although the intensively reared flock is expected to have lower panting score, EOF, and disease burden along with higher growth performance, feed efficiency, and reproductive potential, the literature pertaining to quantification of the differences among the three systems are seldom. Further, as per our knowledge, no related works were ever conducted on Nellore breed of sheep, which plays an indispensable role in the economic growth of South Indian rural livelihood. Hence, this work was conducted to know the effect of three farming systems on the performance, nutrient intake, digestibility coefficients, heat stress indices, disease incidence, and cost economics of Nellore sheep.

## 2. Materials and methods

### 2.1 Study area and ethical statement

The sheep were maintained at Livestock Research Station, Sri Venkateswara Veterinary University (SVVU), Palamaner, Chittoor District, Andhra Pradesh. The region is located at 13.2000° N and 78.7500° E and has an average elevation of 683 meters (2,244 feet). The recordings, observations, and blood collection from the sheep were done with approvals of Institute’s animal ethics committee (IAEC), Sri Venkateswara Veterinary University (SVVU), Tirupati. The guidelines framed by Committee for the Purpose of Control and Supervision of Experiments on Animals (CPCSEA; IV, section 15(1)) of prevention of cruelty to animals (PETA, 1960) were followed sincerely. The animals were housed for further research after completing the trial.

### 2.2 Experimental sheep

The study includes two parallel trials. In the first trial, one hundred and twenty lambs (60 ram-lambs and 60 ewe-lambs; 4-months old) were allotted in a randomized block design under three farming systems viz., extensive, semi-intensive, and intensive systems, such a way that each group received forty lambs (20 ram-lambs and 20 ewe-lambs). The allotted lambs were weaned from their dams maintained under the three rearing systems (S1 File).

Recordings of panting score and EOF values during the three seasons might be influenced by age, thereby causing errors in results. To impede the errors due to age effect, eighteen rams (aged 2 years) were selected for the second trial and allotted to three farming systems, as mentioned earlier. The sheep in intensive group were reared with the floor space area of 1m^2^/animal in the covered shed. Fodder and water arrangements were made available in a hygienic way under zero-grazing system. Provisions for proper feeding and watering were made suitably in this group. In extensive system, the animals were moved into night shelter in the covered shed with a floor space of 1m^2^/animal.

### 2.3 Housing, feeding and health management

The sheep under study were properly identified by ear tagging. Clean and fresh drinking water was provided in the shed throughout the day. In extensive and semi-intensive systems, the sheep were sent for grazing from 8.00 AM to 4.00 PM, while the intensive sheep was offered with fodder (Hybrid Napier) *ad libitum*. The sheep in intensive and semi-intensive systems were provided with concentrate mixture at 1.5% and 1.0% of the body weight, respectively. The concentrate mixture was composed of maize grain, deoiled rice bran, soybean meal, groundnut cake, mineral mixture, and salt at 28.0, 34.0, 25.0, 10.0, 2.0, and 1.0 percent, respectively. The intensive sheep were housed in a well ventilated shed with sun shade.

All the experimental animals were dewormed before starting the experimental trial with broad-spectrum anthelmintic (I.P. 500 mg Niclosamide and 150 mg Albendazole; Vet India Pvt. Ltd.). Deticking was done twice during the experimental period for the control of external parasites with amitraz dip concentrate (I.P. 12.5% w/v; Virbac India Pvt. Ltd.). All the animals were vaccinated against FMD (Indian Immunologicals Pvt. Ltd., India), Enterotoxaemia (Brilliant biopharma Pvt. Ltd., India), Blue tongue (Indian Immunologicals Pvt. Ltd., India), and PPR (Hester Biosciences Ltd., India). Hygienic surroundings were maintained throughout the experimental period.

### 2.4 Recording and sampling

#### 2.4.1 Meteorological parameters

The dry and wet bulb temperatures were recorded using wet and dry-bulb thermometers, respectively, while the relative humidity was measured by using a sling psychrometer. The farm was continuously monitored for these parameters during the entire study period. The rainfall was measured by using rain gauge prepared by calibrated volumetric flask and funnel. The regular datum of wind velocity of the experimental region was collected from www.timeanddate.com. The temperature-humidity index was calculated as per Ravagnolo et al. (2000) [7] as follows;

THI=(1.8×T+32)−{(0.55−0.0055×RH)(1.8×T−26)}

Where, T–Ambient temperature (°C)

RH–Relative Humidity (%)

#### 2.4.2 Heat stress indices

The panting scores were recorded daily at 1400 h during the first four consecutive days of December, May, and August, representing winter, summer, and monsoon seasons, respectively. The procedure used for measuring is as described by Brown-Brandl et al. (2006) [8]. The description of panting scores is provided in supplementary Table (S2 File). About 10 ml of blood was collected in heparinized tubes to ensure the RBC intact. The samples were preserved at -20°C and the EOF test was performed in the lab.

#### 2.4.3 Growth performance

The sheep in trial I were regularly monitored for recording weight gain (WG), average daily gain (ADG), and reproductive parameters throughout the period. The growth trajectory of sheep was described by using the non-linear model (Gompertz curve). A total of 1560 body weight-age recordings (260 per each farming system) were fitted by using gompertz curve as follow;

W(t)=A×exp(−B×exp(−K×t)

Where, W(t) is the body weight of sheep at ‘t’ months of age, A is the asymptotic weight or mature weight, B is constant of integration related with initial weight, K is the maturing rate, and t is the time (month) of growth. The estimations of weight and age at the point of inflection were calculated as;

$$W_{i}=\frac{A}{exp}\;\mathrm{and}\;t_{i}=\frac{ln(B)}{K}$$

The nutrient intakes were recorded by using six rams from each group during the 5^th^ month; hence feed conversion ratio was calculated by using the 5^th^ month body weights as initial recordings and 6^th^ month body weights as final recordings. Initial body weights and body measurements were recorded before the commencement of experiment. The body weights and body measurements of animals in three farming systems were recorded at fortnight intervals before offering the feed and water in the morning. The weights were recorded by weighing the animals using digital balance. The average daily gain (ADG) and feed conversion ratio (FCR) were calculated by using the following formulae;

$$Average\;Daily\;Gain(ADG)=\frac{Final\;weight(kg)-Initial\;weight(kg)}{Number\;of\;days\;of\;growth\;trial}$$

$$Feed\;Conversion\;Ratio(FCR)=\frac{Dry\;matter\;intake(Kg/d)}{Average\;daily\;gain(Kg)}$$

### 2.5 Pasture characteristics and ingestive behavior

The sheep were grazed on pasture in the vicinity of Livestock Research Station (LRS), Palamaner, Andhra Pradesh, India. The pasture botanical composition comprises a mixture of herbaceous plants, grass and browse species, and dominated by *Cynodon dactylon* and *Stylosanthes hemata*. The forage samples (n = 6) were collected from the pastures by means of bite count and hand plucking methods [9,10]. The sheep were acclimatized for an observer for a minimum 15-day period or until the cessation of their movement while the observer was 0.5 to 1.5 meters away. The same observer was used per each sheep to film and monitor the bite count and hand plucking activities. The ingestive behaviour was observed for a continuous 20-second period with 5 minutes interval for 10 hours, i.e., 2 hours a day for five days. The 20-seconds time interval is adequately long to contain several bites and sufficiently short to avoid grouping bites [9]. The observations for 20-second intervals were collected, pooled, and calculated per minute.

Instantaneous intake rates (IIR) were calculated as;

IIR(gDM/min)=IBM×IBF

Where, IBM is instantaneous bite mass (g DM) and IBF is instantaneous bite frequency (bite/min).

The proportions of individual plants were calculated by using instantaneous intake rates over the observed period. The vegetation collected was preserved for estimation of DM and CP contents. To create the plant-database, the type of vegetation was initially identified by ‘PlantNet’ android application and later confirmed by a botanist at Sri Venkateswara University.

### 2.6 Intake and digestibility coefficients

The digestibility coefficients of dry matter (DM) and crude protein (CP) were estimated by using indicator method. Two grams of chromium were given in capsules form (each capsule comprising 1 gm chromium) to the sheep at equal doses in the morning (8:00) and evening (16:00) periods. Faecal grabs were collected directly from anus thrice a day for eight days. Chromium analysis of the fecal samples was done by using the Atomic Absorption Spectrphotometer (GBC Scientific Equipment Pvt. Ltd. Dandenong, Australia) as per the protocol given by Yiakoulaki et al. (1997) [11]. The wavelength, slit width, working range, and sensitivity of the chromium lamp were 357.9 nm, 0.2 nm, 2–15 μg/ml, and 0.05 μg/ml, respectively. The CP digestibility coefficient was determined by using indicator method with chromic oxide as an external indicator [12]. The coefficients are estimated by using the change in the ratio of each nutrient with reference to the chromic oxide concentration in feed and feces. The fecal excretion, dry matter intake, digestibility coefficients of CP are calculated by using the following equations [12];

$$Fecal\;excretion=\frac{Indicator\;amount\;of\;daily\;intake(g/day)}{Indicator\;amount\;of\;feces(g/g\;DM)}$$

$$Dry\;matter\;intake=\frac{Amount\;of\;daily\;fecal\;output(g)}{1-Invitro\;DM\;digestibility}$$

$$Dig.coefficient\;of\;CP=100-100\times \frac{\%\;indicator\;in\;feed\times \%\;CP\;in\;feces}{\%\;indicator\;in\;feces\times \%\;CP\;in\;feed}$$

The % CP in feed for semi-intensive and extensive systems was calculated by using the equations;

$$\mathrm{For}\;\mathrm{semi}‐\mathrm{intensive}\;\mathrm{system}‐\%CP_{\mathrm{feed}}=\Sigma \left(\%CP_{\mathrm{vegetation}}\times PR\right)+CP_{cm}$$

$$\mathrm{For}\;\mathrm{extensive}\;\mathrm{system}‐\%CP_{\mathrm{feed}}=\Sigma \left(\%CP_{\mathrm{vegetation}}\times PR\right)$$

Whereas, CP_vegetation_—CP content of the individual vegetation

PR—Proportion of the individual vegetation

CP_cm_−CP content of the concentrate mixture

### 2.7 Laboratory analyses

Samples of grass, fodder, concentrate mixture, and feces were analyzed for DM and CP, according to AOAC (2007) protocols [13]. The fresh samples were incubated overnight in a hot air oven with 100 ± 5°C. The crude protein was estimated by multiplying the nitrogen content with 6.25. Nitrogen analysis was done by using Turbotherm and Vapodest (Gerhardt, Germany) analyzer. The *in vitro* dry matter digestibility coefficient was determined as per the protocol of Tilley and Terry (1963) [14].

The EOF test was performed as per the protocol of Meamarbashi and Rajabi (2013) [15]. About 3 ml of the blood was transferred into a vial. After centrifugation, 20 μL of packed RBC was transferred to tubes containing distilled water and sodium chloride solutions at 0.45% and 0.9% concentrations, respectively. The three tubes were incubated at 37°C for 30 min, followed by centrifugation at 1300 g for 5 minutes. The hemolysis percentage was calculated at 540 nm in photoelectric colorimeter. The supernatant layer was considered as hemolysed RBC. The percentage of hemolysis in 0.45% and 0.90% saline were calculated by assuming the hemolysis in distilled water as 100%.

### 2.8 Reproductive parameters

The date of puberty (days) was recorded by examining the first estrus behavior by the female hoggets using rams. The rams were introduced into the flock and even allowed for grazing along with ewes in extensive and semi-intensive systems. Ewes being receptive to ram and standing for mounting by ram were considered to be in oestrus. After arriving at the exact date of puberty, the ewes were weighed on the next day before sending for grazing.

The sexual behaviour of the ewes was examined by introducing teaser ram. The ram was introduced at three hours interval to find out the time of cessation of receptive behaviour by the ewes. The behavioural signs observed to know the receptive behaviour include sniffing scrotum and genital areas, soliciting, non-firm standing, head turning, tail fanning, and squatting. Conception percent is the percent of the number of ewes conceived among the number of ewes tupped.

$$Conception\;percent=\frac{Total\;number\;of\;ewes\;conceived}{Total\;number\;of\;ewes\;tupped}\times 100$$

Gestation period was calculated as the duration between the date of conception and date of lambing. The weights were taken before 48 hours of lambing in case of ewes and immediately after birth in case of lambs using electronic balance after correcting the error.

### 2.9 Disease incidence

The sheep managed under the three rearing systems were carefully evaluated for health problems such as diarrhea, bloat, pneumonia, anorexia, pregnancy toxaemia, abscess, foot rot, and tick infestation. Diseases noticed, if any, were noted separately according to farming system and season. The specific diseases were diagnosed according to the symptoms provided in supplementary file 3 (S3 File).

### 2.10 Economic analysis

The cost of fodder, salary paid to labor, veterinary aid, and miscellaneous expenditure and cost of initial lambs were calculated as per the prevailing charges. Similarly, the depreciation and interest percent was calculated according to the current prices. The total cost was calculated by adding total working costs and fixed costs. The notional value of sheep was remunerated to their market value. The total manure output (in tonnes) was derived by using the digestibility and intake values during the digestibility trial period. Miscellaneous income includes the revenue generated by the sale of feed, gunny bags, skins, etc. The net income was calculated by subtracting gross income from total cost. The returns per rupee of expenditure were obtained by considering the net income and initial expenditure.

### 2.11 Statistical analysis

All the recordings of parametric variables were tested for normal distribution by using Kolmogorov-Smirnov test. The recordings of WG and ADG were subjected to multivariate analysis through General Linear Model (GLM) procedure. The initial recordings were included in the model as covariates. The sheep was used as a random effect and the sex and interactions among farming system (FS) and sex (S) were used as fixed effects. The intakes, nutrient digestibilities, FCR, and reproductive parameters of ewes were analyzed by using one-way ANOVA. For EOF, the data were analyzed for statistical difference through GLM univariate procedure. The sheep is used as a random effect and the season and interactions among farming system (FS) and season (S) were used as fixed effects. Post hoc analysis, wherever necessary, is performed by adjusting the data as per the Bonferroni corrections and Tukey’s HSD. The values were presented as means with standard error of means. The panting scores (ordinal data) with reference to feeding system, season, and hour were analyzed for differences according to Kruskal Wallis H test. The *P* values less than 0.05 is considered as significant and those between 0.05 and 0.1 was considered as a trend. Entire statistical analysis was performed by using SPSS version 23.0 [16]. The data of body weight and age were fitted to Gompertz curve-Newton iterative method by using NLIN procedure of SAS Version 9.4 [17]. The correlogram of the data pertaining to heat stress indices and meteorological parameters was prepared by using the ‘corrplot’ function of R (3.6.3 version). All graphs, except correlogram, were generated by using Graph pad Prism Version 7.0.

## 3. Results

### 3.1 Meteorological parameters

**Fig 1:**
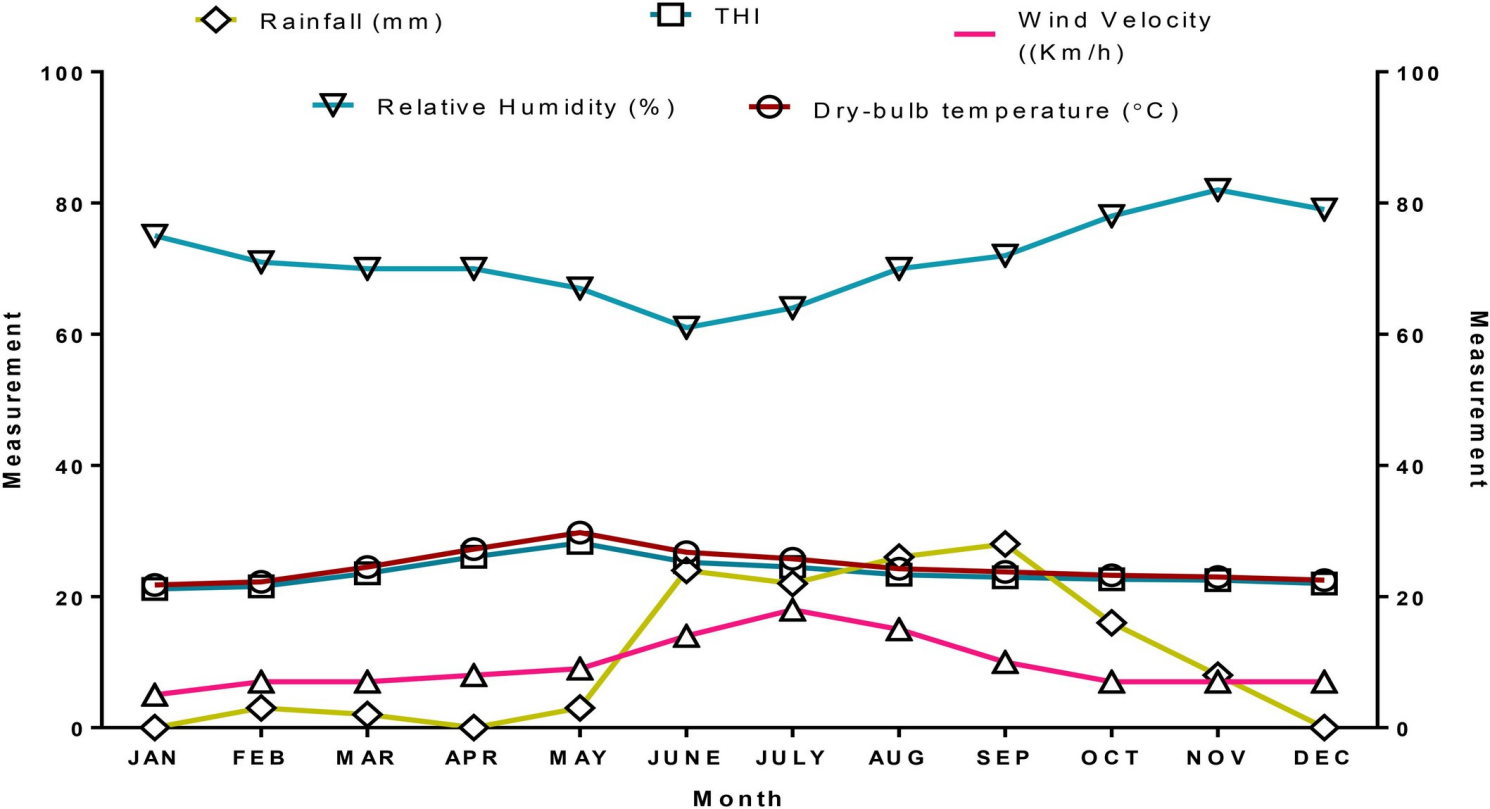
Meteorological data recorded during the experimental study at LFC, Palamaner.

The meteorological data of the experimental region are depicted in Fig 1. The range of dry-bulb temperature (°C), relative humidity (%), wind velocity (km/h), rainfall (mm), and THI of the entire year were 21.75–29.75, 61–82, 7–13, 0–28 and 21.18–28.18, respectively. The dry-bulb temperature and THI were highest in May and lowest in January.

### 3.2 Heat stress indices

**Fig 2:**
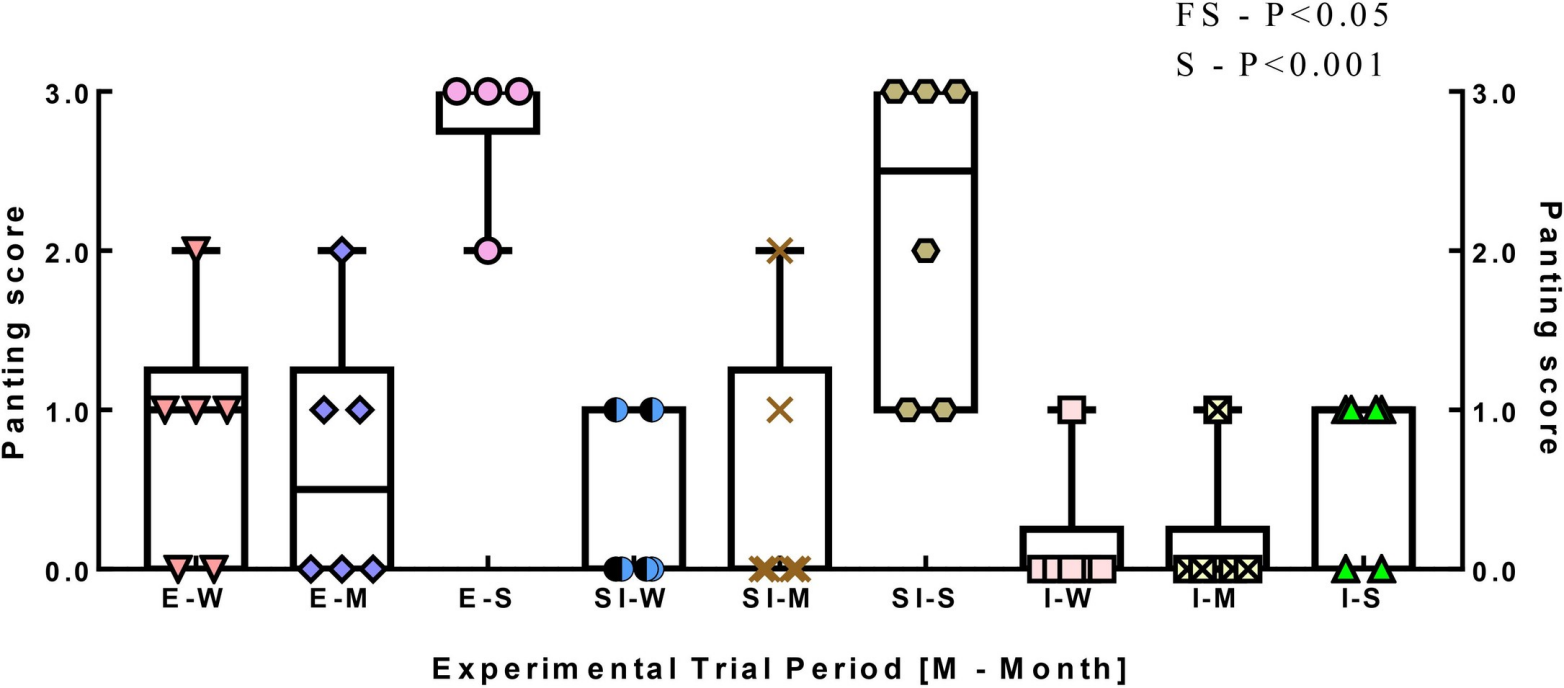
Box and whisker plot of panting score of sheep with reference to farming system and season. E-W, Extensive-Winter; E-M, Extensive-Monsoon; E-S, Extensive-Summer; SI-W, Semi-intensive-Winter; SI-M, Semi-intensive-Monsoon; SI-S, Semi-intensive-Summer; I-W, Intensive-Winter; I-M, Intensive-Monsoon; I-S, Intensive-Summer.

**Fig 3:**
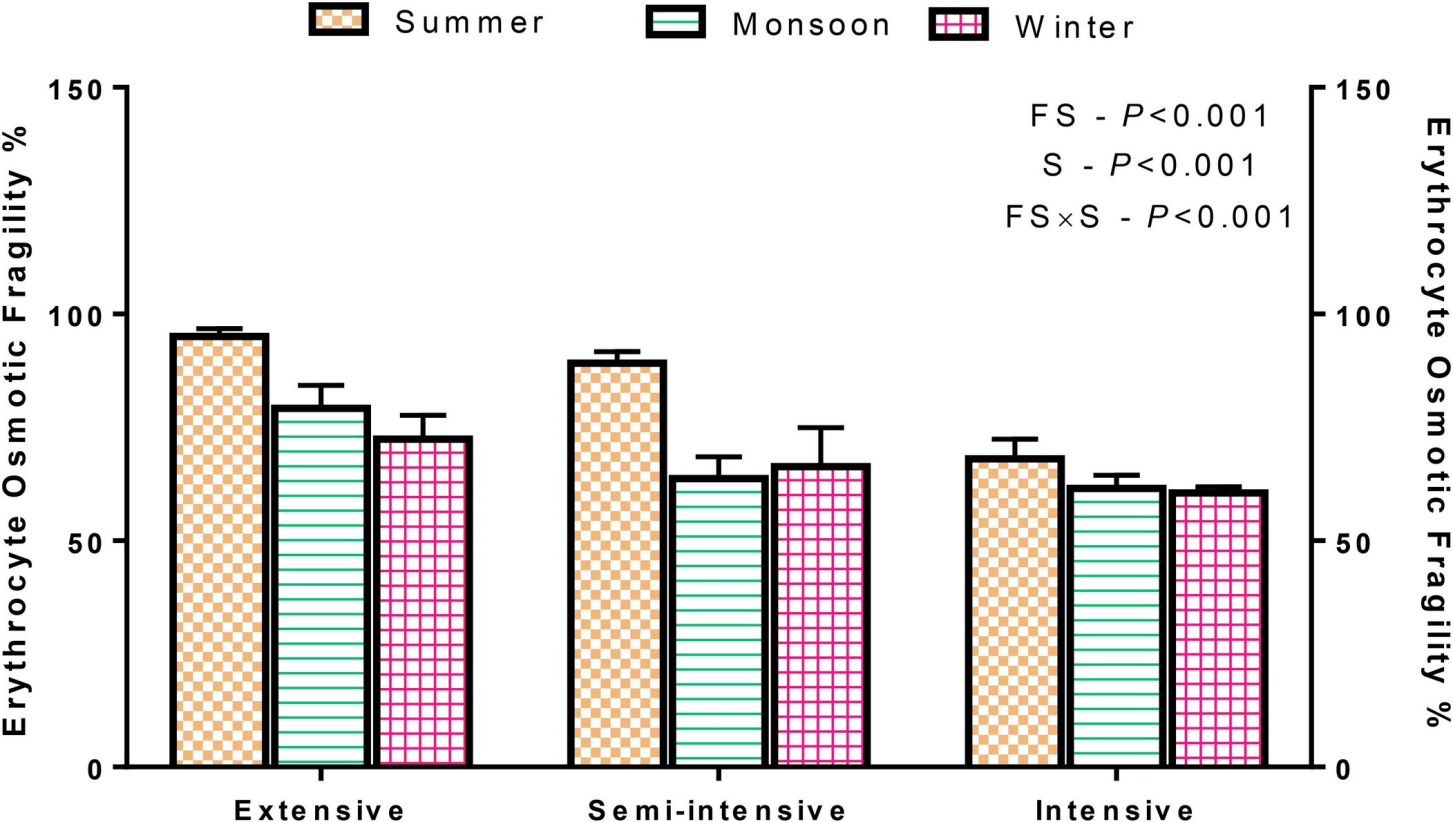
Erythrocyte osmotic fragility (%) of the sheep reared under different farming systems. E-W, Extensive-Winter; E-M, Extensive-Monsoon; E-S, Extensive-Summer; SI-W, Semi-intensive-Winter; SI-M, Semi-intensive-Monsoon; SI-S, Semi-intensive-Summer; I-W, Intensive-Winter; I-M, Intensive-Monsoon; I-S, Intensive-Summer.

The box and whisker plot for panting scores showed that the median values of panting scores were higher (P<0.05) in sheep managed in extensive and semi-intensive farming systems (Fig 2). Seasonal influence was also noticed with the higher (P<0.001) scores in summer season. The practice of grazing in extensive and semi-intensive feeding systems increased (P<0.001) the EOF percent (Fig 3). Similarly, the seasonal effect was noticed with higher (P<0.001) EOF in summer season. Further, a feeding system × season interaction (P<0.001) was observed for EOF test.

**Fig 4:**
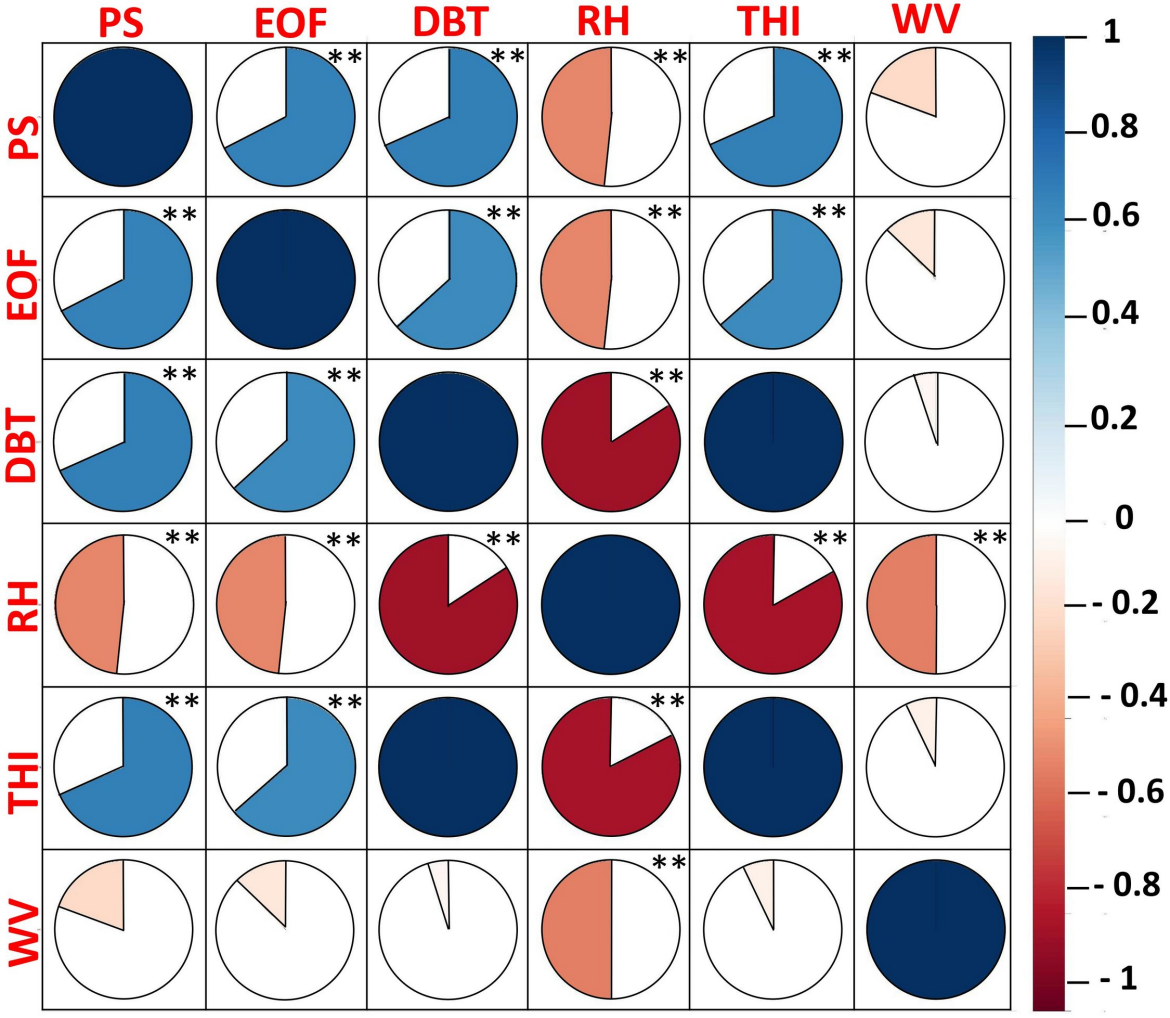
Correlogram of heat stress indices and meteorological parameters (**P<0.01). PS, Panting score; EOF, Erythrocyte osmotic fragility; DBT, Dry bulb temperature; RH, Relative humidity; THI, Temperature humidity index; RF, Rain fall; WV, Wind velocity.

The correlogram of heat stress indices and meteorological parameters showed that panting score and EOF were positively correlated (P<0.01) to the dry-bulb temperature and THI and negatively correlated (P<0.01) to the relative humidity (Fig 4). The relative humidity is inversely correlated to THI and dry-bulb temperature. Among the meteoreological parameters, no significant relationship was found for heat stress indices and wind velocity.

### 3.3 Growth performance

The recorded monthly body weight changes and average daily gains are presented in Table 1. The weight gain of the sheep was higher (P<0.001) in intensive system followed by semi-intensive and extensive system. Males showed higher (P<0.001) weight gains in all the growth periods, except for 13–15 months. Further, interactions were noticed between farming systems and sex, for all the growth periods, except during 4–6 and 13–15 months. The ADG (g) was higher in intensively reared sheep irrespective of the growth periods. Significant interactions were noticed for farming systems and sex during all the periods, except for 13 to 15 months period. The monthly body weight changes as a function of age and different rearing systems are presented in supplementary file 4 (S4 File). The growth curve patterns of Nellore sheep are presented in Table 2. The asymptotic weights were higher for sheep reared under intensive farming system, followed by semi-intensive and intensive systems. The estimated parameters of A and B were higher for males compared to females. The maturation rates were lower for intensive sheep; however, the weight of inflection did not follow any distinctive pattern.

**Table 1:** Weight gain and Average daily gain of the sheep reared under different farming systems.

Period (Months)	Farming system			Sex		SEM	*P* values		
	EXT	SI	INT	Male	Female		FS	S	FS×S
Weight gain (kg)									
4–6	7.02^a^	9.26^b^	11.09^c^	10.40	7.84	0.27	<0.001	<0.001	0.073
7–9	3.85^a^	4.33^b^	7.93^c^	6.13	4.61	0.19	<0.001	<0.001	0.003
10–12	2.86^a^	3.34^b^	5.12^c^	4.26	3.29	0.14	<0.001	<0.001	<0.001
13–15	1.87^a^	2.37^b^	2.89^c^	2.48	2.27	0.15	<0.001	0.119	0.989
4–15	15.60^a^	19.30^b^	27.02^c^	23.27	18.01	0.41	<0.001	<0.001	0.003
Average daily gain (g)									
4–6	77.14^a^	101.73^b^	121.84^c^	114.30	86.17	2.99	<0.001	<0.001	0.073
7–9	42.34^a^	47.61^b^	87.14^c^	67.38	50.68	2.09	<0.001	<0.001	0.003
10–12	31.43^a^	36.70^b^	56.21^c^	46.76	36.14	1.50	<0.001	<0.001	<0.001
13–15	20.49^a^	26.02^b^	31.79^c^	27.29	24.91	1.68	<0.001	0.119	0.989
4–15	42.85^a^	53.02^b^	74.24^c^	63.93	49.47	1.11	<0.001	<0.001	0.003

Ext—Extensive, SI–Semi intensive, Int–Intensive, SEM–Standard error of mean, FS–Farming system, S–Sex, *P* Val–*P* values.

**Table 2:** Estimates of growth curve parameters for sheep reared under different farming systems.

Rearing system-Sex	A	B	K	W_i_	t_i_
Extensive-Male	32.69	2.55	0.26	12.03	3.60
Extensive-Female	29.43	2.49	0.27	10.83	3.38
Semi-intensive-Male	37.84	2.17	0.21	13.92	3.69
Semi-Intensive-Female	34.57	1.78	0.23	12.72	2.51
Intensive-Male	46.67	3.05	0.20	17.17	5.58
Intensive-Female	43.21	1.98	0.22	15.90	3.10
SEM	0.71	0.16	0.02	0.98	0.42

A, Asymptotic weight or mature weight (kg); B, Integration constant related to initial animal weight (kg); K, Maturation rate; W_i_, Weight at inflection (kg); t_i_, Time at inflection (month).

### 3.4 Ingestive behavior

The ingestive behaviour of the sheep is presented in Table 3. The IBM was higher for *Amaranthus viridis*, whereas higher IBF was found for *Cyanodon dactylon*. However, the sheep spent higher time while browsing the *Stylo hemata* compared to other plant species. Accordingly, the total DMI and proportion of vegetation was highest for *Stylo hemata* followed by *Cynodon dactylon* and *Tridax procumbens* species. The sheep showed least interest for *Tribulus terrestris* and *Lantana camara* species.

**Table 3:** Ingestive behavior of the grazing sheep.

S.No.	Name of browse	IBM	IBF	IIR	TSG	DMI	Proportion
1	*Stylo hemata*	0.138	25.00	3.45	362.00	20.82	13.03
2	*Cyanodon dactylon*	0.113	29.00	3.28	302.00	16.49	10.33
3	*Tridax procumbens*	0.177	20.00	3.54	292.00	17.23	10.79
4	*Euphorbia hirta*	0.139	22.00	3.06	262.00	13.35	8.36
5	*Amaranthus viridis*	0.157	24.00	3.77	195.00	12.25	7.67
6	*Commelina benghalensis*	0.186	18.00	3.35	181.00	10.10	6.32
7	*Acalypha indica*	0.141	18.00	2.54	185.00	7.83	4.90
8	*Boerhavia diffusa*	0.154	17.00	2.62	175.00	7.64	4.78
9	*Sida acuta*	0.145	18.00	2.61	166.00	7.22	4.52
10	*Tephrosia purpurea*	0.134	19.00	2.55	160.00	6.79	4.25
11	*Tribulus terrestris*	0.155	24.00	3.72	56.00	3.47	2.17
12	*Lantana camara*	0.164	19.00	3.12	64.00	3.32	2.08

IBM, Instantaneous bite mass (g DM); IBF, Instantaneous bite frequency (bite/min); IIR, Instantaneous intake rates (g DM/min); TSG, Total seconds grazed on browse; DMI, Total DM intake (g).

### 3.5 Intake and digestibility coefficients

The DM intake, nutrient digestibility coefficients, and FCR of the sheep reared under three farming systems are presented in Table 4. The intensive sheep showed higher (P<0.05) DMI in terms of grams and percent BW. The IVDMD and CP digestibility were higher (P<0.01) intensive sheep compared to those reared under other systems. Further, sheep maintained under zero-grazing showed better (P<0.05) FCR.

**Table 4:** Digestibility parameters and reproductive performance of the sheep under different farming systems.

Parameter	Farming system			SEM	*P* value
	EXT	SI	INT		
Intake and nutrient digestibility coefficients*					
DMI (g/d)	612.91^a^	716.83^b^	733.32^c^	38.01	0.045
DMI (%BW)	3.35^a^	3.70^b^	4.25^c^	0.16	0.034
IVDMD*	51.24^a^	63.5^b^	74.2^c^	1.25	<0.001
CP dig	46.35^a^	59.76^b^	66.1^c^	3.25	0.002
FCR	10.84^b^	8.92^b^	7.44^a^	0.98	0.041
Reproductive performance					
Age at puberty (days)	323.70^c^	312.05^b^	281.90^a^	3.73	<0.001
Weight at puberty (kg)	26.65	27.70	27.91	0.38	0.058
Estrus cycle length (days)	17.45	17.65	17.55	0.24	0.838
Estrus duration (hours)	25.95	24.95	25.75	0.34	0.114
Conception percent (%)	70.00	70.00	80.00	0.10	0.722
Gestation period (days)	148.08	150.07	151.00	1.09	0.643
Lambing percent (%)	65.00	75.00	80.00	0.10	0.564
Birth wt. (kg)	2.15^a^	2.41^a^	2.82^b^	0.16	<0.001

*Mean values and standard error mean of *in vitro* digestibilities (triplicates).

### 3.6 Reproductive parameters of ewes

No significant differences were observed for estrus cycle length, estrus duration, conception percent, gestation period, and lambing percent (Table 4). The weight at puberty tended to be significant (P = 0.058) among the three rearing groups. The age at puberty was lower (P<0.001) while the birth weight was higher (P<0.001) in intensively reared sheep.

### 3.7 Disease incidence

**Fig 5:**
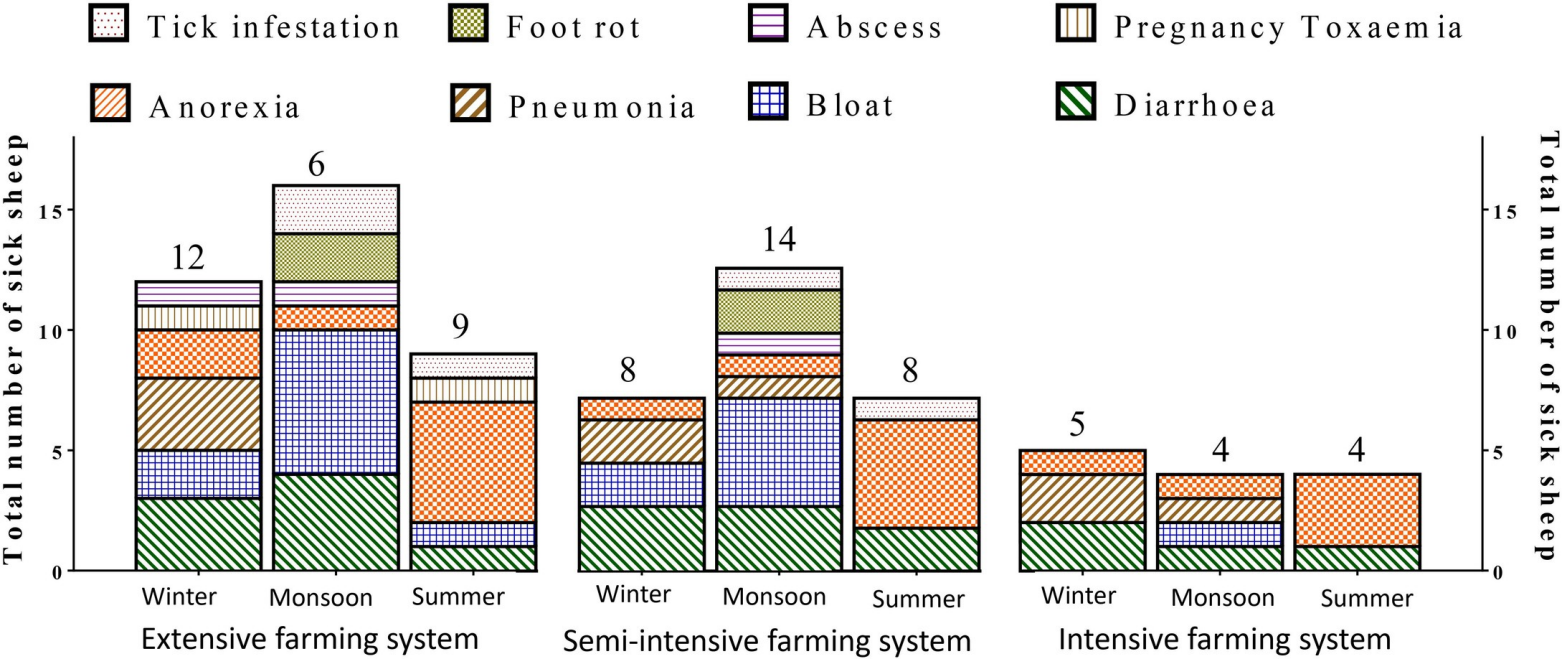
Disease incidence of sheep with reference to farming system and season.

Highest number of disease cases were observed in extensive system of rearing followed by semi-intensive and intensive systems. Regarding the seasons, the disease incidence was highest in rainy and winter seasons compared to summer season (Fig 5).

### 3.8 Economic analysis

The capital and recurring expenditure and total income generated from the 40-sized sheep unit under the three farming systems are presented in Table 5. The initial cost for purchasing ewes and rams, depreciation, and interest were same for the three rearing systems. The total cost required for setting extensive, semi-intensive, and intensive rearing units with 40-sheep capacity was Rs. 3,03,730/-, 3,77,618/-, and 4,68,225/-, respectively. Concentrate and manpower were responsible for most of the production cost. The income generated through selling manure was higher in intensive system, while the miscellaneous income was same for the three systems. The gross income and net income were higher for intensive system, whereas the returns per rupee of expenditure are higher for extensive system of rearing.

**Table 5:** Economic analysis of sheep production under different farming systems.

Total working cost	Farming system		
	Extensive	Semi-intensive	Intensive
A. Capital expenditure			
Value of animals	1,50,000 (49.38)	1,50,000 (39.72)	1,50,000 (32.04)
Depreciation	4,920 (1.62)	4,920 (1.30)	4,920 (1.05)
Interest	12,600 (4.15)	12,600 (3.17)	12,600 (2.68)
Total fixed cost	1,67,520 (55.16)	1,67,520 (44.36)	1,67,520 (35.77)
B. Recurring expenditure			
Fodder	0	0	44,930 (9.60)
Concentrates	0	69,734 (18.47)	1,10,283 (23.55)
Man power	96,000 (31.60)	96,000 (25.42)	96,000 (20.50)
Veterinary aid	2,000 (0.66)	2,000 (0.52)	2,000 (0.43)
Maintenance cost	30,000 (9.87)	30,000 (7.94)	30,000 (6.41)
Miscellaneous	500 (0.16)	500 (0.14)	500 (0.11)
Interest on capital	7,710 (2.54)	11,864 (3.15)	16,992 (3.63)
Total working cost	1,36,210 (44.84)	2,10,098 (55.64)	3,00,705 (64.23)
C. Total cost (A+B)	3,03,730 (100)	3,77,618 (100)	4,68,225 (100)
D. Income			
Notional value of rams	1,60,000	1,82,050	2,14,350
Notional value of ewes	1,31,985	1,48,050	1,61,730
Total value of animals	2,91,985	3,30,100	3,76,080
Sale of manure	3,000	4,500	9,000
Miscellaneous Income	1,500	1,500	1,500
Gross Income	5,88,470	6,66,200	7,62,660
Net Income	2,84,740	2,88,582	2,94,435
Returns per rupee of expenditure	1.93	1.76	1.63

Values are in Indian rupees (1 US dollar = 76.50 INR).

Proportion of each cost item was shown in parenthesis.

## 4. Discussion

### 4.1 Meteorological parameters

The severity of heat stress was estimated by calculating the THI, which considered both ambient temperature and relative humidity of the experimental farm. The dry-bulb temperature and THI from April to June were within the upper critical zone, suggesting that the sheep under semi-intensive or extensive systems of management experienced thermal stress in these months. During heat-stressed seasons, the sheep undergo some physiological changes as a method of adaptation [18]. Within the day of recording, the THI was higher during afternoon hours and lower in morning periods revealing the heat stress during noon hours. The mean THI recorded in the summer, the hottest season of the year, indicated the possibility of moderate stress in the rams.

### 4.2 Heat stress indices and correlogram

Higher panting scores were recorded from the sheep grazing outside compared to those reared under intensive farming systems. Both seasonal influence and its interactions with farming systems were also noticed with higher scores in summer season. Similar relation of panting score with heat stress was proposed by several author [6,19–21]. Panting score is a measure of respiratory rate and body temperature. In heat-stressed sheep, the changes in respiratory dynamics could be categorized into two phases of panting [22]. The first phase is characterized by a rapid shallow panting phase (score 0 to 2), which was noticed in the extensive and semi-intensive sheep during winter and monsoon periods. However, the summer season caused second phase scores characterized by deeper respirations and open-mouthed panting (score 2 to 4).

The sheep reared in extensive and semi-intensive farming systems showed higher EOF due to the higher THI. Similar reports were reported elsewhere [21,23]. The stress caused by increased body temperature may enhance the production of free radicals, thereby causing lipid peroxidation. The lipid peroxidation increases the fragility of RBC membrane, consequently increasing the EOF [24,25]. During heat stress periods, the high panting score led- respiratory alkalosis will increase the fragility of RBC, thereby increasing the EOF [6]. Few authors reported the increased EOF even in extreme cold climatic seasons [26] or transport stress [25], revealing that the EOF could be increased in stress, irrespective of the type.

As anticipated, the correlogram displayed positive correlations among panting score, EOF, dry-bulb temperature, and THI and inverse relation of the relative humidity to heat stress indices. As discussed above, the phenomenon of increased respiratory rate, body temperature, and fragility of RBC during heat stress explains these positive correlations. Our study revealed no linear relation between heat stress indices and wind velocity. In corroboration, Papanastasiou and Bartzanas (2014) [27] found that wind speed was not a strong factor in influencing heat-stress levels. On the contrary, Reddy et al. (2019) [21] revealed a significant negative correlation between heat stress indices and wind speed [6]. The authors revealed that wind speed possesses an ameliorating effect on the higher THI. These alterations might be attributed to the methodology differences in experiments. The negative factor in the methodology of our study is the adoption of website data for wind speed of the region, instead of direct measurements within the experimental farm.

### 4.3 Growth performance

The initial body weights of the lambs were different because of the variation in their dams’ rearing systems. Body weight gains are highly correlated to certain biometric characteristics; hence, considered as the most important economic trait of sheep [28]. The body weight gains were higher in intensive sheep because of the high plane of nutrition and nutrient intake. The limited stress factors in intensive rearing is another important reason for the higher weight gains. Besides, the energy sparing effect because of the absence of wandering might have improved the condition of the sheep. Similar results were reported by several authors [29–31]. However, the results from Singh et al. (2003) [32] showed no significant differences in body weight gains of sheep reared in extensive and semi-intensive systems [32]. The age and sex had a significant influence on the growth rate, which was higher in males compared to females, irrespective of the farming system. Similar results were reported elsewhere [33,34]. The higher weight gains in ram lambs compared to ewe lambs during all stages of growth might be due to the quantitative differences in the secretion of growth and sex hormones [30]. The lower ADG in sheep reared under extensive and semi-intensive systems might be due to decreased feed intake because of stress. Wandering under daylight in these systems could cause heat stress, which increases water intake, consequently reducing feed intake. These results were in close agreement with the reports of few authors [35–37].

However, Singh et al. (2003) [32] revealed no differences of ADG between semi-intensively and intensively reared sheep [32]. Few studies reported higher ADG in semi-intensively reared sheep compared to those maintained under intensive system; however, the nutrient content of the diet fed in intensive system is lower compared to the present study [38]. In all the three farming systems, the lambs had higher ADG during 4–6 months period and a linear decrease was observed with increased age (7–9 m, 10–12 m, and 13–15 m). The higher ADG recorded during 4–6 month period could be due to the efficient utilization of feed resources at the particular age group. Furthermore, the interactions between farming system and sex revealed a higher ADG in intensive male lambs and lower ADG in extensive female lambs.

The higher asymptotic weight in males compared to females imply possible evidences on the impact of sexual dimorphism on weight gain. The male lambs had higher estimated mature weight than female lambs, which may be explained by slower maturity rate [39]. Similar phenomenon in male lambs were found in Kordi [39], Mehraban [40], Shall [41], and Hemsin [42] sheep breeds. The integration constant (B) did not show any clear interpretation, as mentioned by Malhado et al. (2009) [43]. The maturation rates were higher for female sheep compared to males. In this view, these results share similarities with the studies in Hemsin [42] and Horro [44] sheep breeds.

### 4.4 Ingestive behavior

Although the IIR (g DM/min) was higher for Euphorbia hirta, the sheep consumed *Stylo hemata* at higher quantities, followed by *Cyanodon dactylon*. It is of no surprise to obtain these results, as the pastureland is dominated by these two species, which were sown earlier under pasture establishment program. The sheep were least grazed on *Tribulus terrestris* and *Lantana camara* species. Fascinatingly, the literature revealed photosensitization effect of the two plants evidencing the self-selection behaviour of sheep [45,46].

Despite not enumerated, visual inspection of the rangeland showed higher proportion of *Tephrosia pupurea*, in terms of biomass and botanical composition. However, the legume was the third least grazed plant among all vegetation. In this context, Rajendran and Balakrishnan (2012) [47] reported that Indian sheep grazing rangelands do not prefer Tephrosia, which is mostly consumed during scarcity periods [47]. Similar to the present study, Agreil et al. (2005) [9] observed a wide range of IBM and IBF in ewes grazed on heterogeneous vegetation [9]. In rangelands, the bite masses and bite frequency depend on type and maturity status of vegetation along with nutritional and motivation state of sheep [48].

### 4.5 Intake and digestibility coefficients

The dry matter intake was higher in intensively reared sheep. Likewise, few studies reported a higher dry matter intake in sheep maintained under intensive system compared to those sent for grazing [49,50]. The literature revealed several possible reasons for the low DMI in extensive and semi-intensive sheep. As argued by Pereira et al. (2008) [51] and Hyder et al. (2017b) [52], exposing sheep to high ambient temperature increases water consumption to combat the increased heat dissipation, consequently reducing the feed intake [51,52]. The higher water intake might be related to the greater water turn over in the body because of evaporation through the respiratory tract and skin [53]. Rana et al. (2014) [54] attributed the decreased dry matter intake in grazing sheep to the reduced rate of passage of digesta [54]. The high moisture content of grasses coupled with scarce and scattered grazing resources decreased the nutrient intake compared to the intensive sheep, which were fed on nutrient-rich ration [55]. Besides, the higher temperature causes reduced blood flow to the rumen and decreases ruminal motility and rumination, thereby depressing the intake [52].

Apart from the intakes, sheep reared under extensive system evidenced lower CP digestibility coefficient and IVDMD, indicating the poor quality of feed. The higher DMI and nutrient digestibility coefficients in intensive sheep representing a high plane of nutrition. The adverse effects of heat stress on nutrient digestibility coefficients in grazing sheep is well evidenced [56,57]. These depressions were particularly predominant on feeding the forage-based feed alone, like in extensive rearing systems [57].

Intensive rearing showed better FCR compared to semi-intensive and extensive rearing. In corroboration, Pardua et al. (1997) [58] reported lower FCR in sheep exposed to hot climatic conditions as compared to those reared under the shed [58]. Later, Patel et al. (2004) [59] reported better FCR in the sheep reared under intensive production systems [59]. The exposure of sheep to high ambient temperature stimulates the peripheral thermal receptors and transmit suppressive nerve impulse to the appetite center of hypothalamus, thus decreasing the feed intake and FCR [57]. Scarce biomass availability, poor nutritive value of feed, and energy losses during grazing are the pivot explanations for the recorded low FCR in extensive system. The lambs under intensive rearing had sufficient time for rumination and regurgitation, thereby aiding in proficient feed conversion into muscle development and hence the bodyweight gains.

### 4.6 Reproductive parameters of ewes

Despite the fact that extensive sheep had low plane of nutrition and high stress, the type of farming system did not influence the weight at puberty, length of estrus cycle, duration of estrus, conception percent, gestation period, and lambing percent. These results are in close agreement with the outcomes of few researchers [33,60,61]. However, Naqvi et al. (2001) [62] reported favorable response on estrus percentage, estrus duration, onset of estrus, and ovulation response in sheep supplemented with concentrate in intensive system [62]. In another study, Berhanu et al. (2013) [63] observed an increased conception percent up to 29% in the groups fed with concentrate mixtures compared to the other group sent for grazing alone [63]. Although not significant, the weight at puberty tended to be higher for intensive sheep. The bodyweight of the growing ewes is an indispensable factor for attainment of puberty. Achievement of bodyweight at an earlier age through strategic feeding interventions during the post-weaning period is critical for effectual reproductive performance, which could be achieved by practicing intensive farming. The ewes reared under intensive system matured early, which could be attributed to their faster growth rates. Likewise, Zohara et al. (2014) [64] reported a faster puberty rate of Bangladesh indigenous ewes supplemented with concentrate in intensive farming compared to extensively reared sheep [64]. Further, Chaturvedi et al. (2010) [36] reported less number of days taken to conceive after flushing in flushed ewes as compared to non-flushed ewes [36]. These results share similarities with those of Meenakshi Sundaram (2001) [29] and El-Hag et al. (2007) [65].

The birth weight of lambs born to intensively reared sheep is higher than those reared under the two other systems. Absence of stillbirths in intensive rearing is another reason for higher birth weights. The higher birth weight of lambs in intensive and semi-intensive sheep could be related to the flushing ration [36]. These results are in corroboration to Sejian et al. (2010) [66], who reported that combined stressors (heat and nutritional) significantly reduced birth weight of lambs [66].

### 4.7 Disease incidence

The disease incidence was higher in extensive rearing system and rainy season compared to other systems and seasons. The study revealed that sending sheep for grazing, especially during rainy season caused more health-related issues. The higher rates of anorexia cases in extensive system and summer season are directly related to heat stress. During heat stress, the efficiency of dry matter intake will be reduced in sheep, consequently causing inappetence and anorexia [18]. Grazing sheep on lush green pastures, especially leguminous pastures, is one of the major causes of bloat [67]. The lush green pastures during rainy season could be related to bloat in sheep grazed on open pastureland. Besides, the incidence of bloat might be aggravated by the presence of leguminous pastures, such as *stylo hemata* in the grazing region.

Most of the causative factors for pneumonia are intensified in winter season compared to summer or autumn seasons [68]. Pregnancy toxaemia is a common metabolic disorder of ewes that is triggered by the high-energy requirements in the later stages of pregnancy being greater than the energy provided by the diet consumed. The sheep reared under extensive system are devoid of concentrate mixture and hence deficient in the required energy, particularly for pregnant ewes. Therefore, the pregnancy toxaemia cases were observed in the ewes reared in extensive system alone. As mentioned by Patel et al. (2013) [61], the tick infestation was higher in rainy and summer seasons compared to winter seasons [61]. The authors reported that the higher tick infestation rates in the respective seasons might be due to the congenial environment for the growth and reproduction of external parasites. However, the prevalence rates of tick infestation may vary with geographical locations and climatic conditions of the experimental region and area [69].

### 4.8 Economic analysis

The cost of manpower used for feeding, watering, and shed cleaning was similar in the three farming systems; however, the cost of concentrate in intensive system was higher compared to semi-intensive system because of the higher allocation percent (1.5% BW Vs. 1.0% BW). Among all the parameters, the cost of concentrate and manpower were higher with a huge margin of difference on comparing to other recurring expenditure factors. The huge cost of concentrate demands the necessity of reducing the concentrate cost by incorporating agro-industrial by-products such as dried distiller grain solubles, urea, salseed meal, neem seed cake, and crop residue-based total mixed rations [21].

For extensive system, the major cost component among the working cost fractions was the human labour with 31.60% of the total cost. Provision of water and electricity constituted about 9.8% of total cost. The manure availability was higher in intensive system compared to extensive or semi-intensive system, as sheep in the latter two systems were sent for grazing, and hence, major chunk of the fecal material was deposited outside. Despite not fed with concentrates or fodder, the lower body weights of sheep under extensive system render the systems less profitable. The higher notional values and more newborn lambs, along with rapid body weights of intensive sheep, made the system more cost-effective with higher gross and net incomes. As explained by Raineri et al. (2015) [70], the gross and net incomes are more related to the zootechnical indexes such as body weights and conception percent rather than the high expenses. However, in view of the small and marginal farmers of developing countries like India, the minimum input systems (extensive or semi-intensive system) could be projected as advantageous farming systems because of the more returns per rupee of expenditure. The aforementioned notion is especially true for the farmers or entrepreneurs with monetary restrictions and limited resources such as land and water supply. According to Mbow et al. (2019) [3], nearly 500 million people practice extensive farming globally, with 75% of countries having pastoral communities [3].

## Conclusion

From the present study, it can be concluded that extensive and semi-intensive farming systems imply heat stress in growing sheep, thereby decreasing the growth rate and bodyweight gains. The limited feed resources in extensive farming system may aggravate the weight loss in sheep. It is also evident that the type of farming system has a bigger influence on the quantity and quality of feed intake. The study revealed that the existing extensive rearing system in India could lead to enormous losses of small ruminant’s production ability. Gompertz curve parameters facilitates the validation of non-linear models in selecting the best performing animals under different managing systems. The intensive or semi-intensive rearing systems may extend lifetime productivity by enhancing reproductive performance of ewes. Intensive sheep evidenced higher gross and net incomes; however, the extensive farming system is proposed as a low-cost business idea for entrepreneurs with inadequate initial capital. Nevertheless, the higher disease incidence, panting scores, and erythrocyte osmotic fragilities reveal the compromised animal welfare in extensive and semi-intensive systems.

## Supporting information

S1 File Initial body weights (kg) of the lambs. 1a. Initial body weights (kg) of lambs allotted to intensive system. 1b. Initial body weights (kg) of lambs allotted to semi-intensive system. 1c. Initial body weights (kg) of lambs allotted to extensive system.
(PDF)

S2 File Description of panting score. (PDF)

S3 File Symptoms for diagnosing the diseases of sheep reared under different farming systems. (PDF)

S4 File Body weight changes with reference to the farming system. (PDF)

## Acronyms

A
ADG
B
BW
CP
CPm
DBT
DM: Dry matter
DMI: Dry matter intake
E-M: Extensive-Monsoon
EOF: Erythrocyte osmotic fragility
E-S: Extensive-Summer
E-W: Extensive-Winter
Ext: Extensive system
FCR: Feed conversion ratio
FS: Feeding system
IBF: Instantaneous bite frequency
IBM: Instantaneous bite mass
IIR: Instantaneous intake rates
I-M: Intensive-Monsoon
Int: Intensive system
I-S: Intensive-Summer
IVDMD: *In vitro* dry matter digestibility
I-W: Intensive-Winter
K: Maturing rate
LRS: Livestock Research Station
PS: Panting score
PS: Panting score
RBC: Red blood cells
RF: Rain fall
RH: Relative Humidity
RH: Relative humidity
S: Season
SI: Semi-intensive system
SI-M: Semi-intensive-Monsoon
SI-S: Semi-intensive-Summer
SI-W: Semi-intensive-Winter
T: Ambient temperature
t: time (month) of growth
THI: Temperature humidity index
TSG: Total seconds grazed on browse
WG: Weight gain
WV: Wind velocity
