# Supplementary material for: Farming systems in sheep rearing: Impact on growth and reproductive performance, nutrient digestibility, disease incidence and heat stress indices
Source: PLoS One. 2021 Jan 13;16(1):e0244922. doi: 10.1371/journal.pone.0244922 (PMC7806139; doi:10.1371/journal.pone.0244922)
Supplement: S1 File — 1a. Initial body weights (kg) of lambs allotted to intensive system. 1b. Initial body weights (kg) of lambs allotted to semi-intensive system. 1c. Initial body weights (kg) of lambs allotted to extensive system. (PDF) [file pone.0244922.s001.pdf]

Supplementary file 1. Initial Body weights (kg) of the lambs

1a. Initial body weights (kg) of lambs allotted to intensive system

Animal No. (male)	**Intensive System**		Animal No. (Female)
	**Male**	**Female**	
1	16	18	21
2	17	17.6	22
3	16.8	17	23
4	12.4	15	24
5	16.4	16.5	25
6	14.6	17	26
7	14.6	16	27
8	18	17	28
9	12	15	29
10	13.6	16.6	30
11	11.6	17.3	31
12	10.8	14.2	32
13	12.4	17	33
14	12	13.5	34
15	12.4	13.8	35
16	16.4	15.5	36
17	16	15.8	37
18	14	13.3	38
19	17.6	18.2	39
20	11	17	40
**Average**	**14.28**	**16.07**	**Average**
**SEM**	**0.53**	**0.33**	**SEM**

1b. Initial body weights (kg) of lambs allotted to semi-intensive system

Animal No. (male)	Semi-intensive System		Animal No. (Female)
	**Male**	**Female**	
41	14	15	61
42	12	12.5	62
43	15.8	15.5	63
44	13.8	18	64
45	15.4	17.8	65
46	15.5	18	66
47	15	16.5	67
48	17	17	68
49	14.7	16.5	69
50	14.9	15.6	70
51	17	15.6	71
52	14.5	15.4	72
53	14.8	14	73
54	18.6	15.8	74
55	15.8	15	75
56	14.8	16.4	76
57	14.4	15	77
58	18.9	14	78
59	11.5	16	79
60	13	13.2	80
**Average**	**15.07**	**15.64**	**Average**
**SEM**	**0.42**	**0.33**	**SEM**

1c. Initial body weights (kg) of lambs allotted to extensive system

Animal No. (male)	Extensive System		Animal No. (Female)
	**Male**	**Female**	
81	13.6	14.52	101
82	16.8	9.2	102
83	12.4	12.85	103
84	13.6	9.3	104
85	9	12.4	105
86	10.6	12.2	106
87	13	12	107
88	12.2	14	108
89	9.8	14.4	109
90	10.8	12	110
91	14.6	12.8	111
92	10	10.54	112
93	11.8	12	113
94	15.8	13.6	114
95	9.2	11.8	115
96	11.3	12	116
97	10.6	14.5	117
98	10.5	13	118
99	16	14	119
100	14.8	11.5	120
**Average**	**12.32**	**12.43**	**Average**
**SEM**	**0.53**	**0.35**	**SEM**
