# Supplementary material for: Farming systems in sheep rearing: Impact on growth and reproductive performance, nutrient digestibility, disease incidence and heat stress indices
Source: PLoS One. 2021 Jan 13;16(1):e0244922. doi: 10.1371/journal.pone.0244922 (PMC7806139; doi:10.1371/journal.pone.0244922)
Supplement: S2 File — (PDF) [file pone.0244922.s002.pdf]

Supplementary File 2 Description of panting score

Score	Breathing pattern	Description of panting
0	60 or fewer breaths/min	Normal respiration
1	60-90 breaths/min	Slightly elevated respiration
2	90-120 breaths/min	Moderate panting and/or the presence of drool or a small amount of saliva,
3	120-150 breaths/min	Heavy open-mouthed panting; saliva usually present
4	>150 breaths/min	Severe open-mouthed panting accompanied by protruding tongue and excessive salivation
