# Supplementary material for: Farming systems in sheep rearing: Impact on growth and reproductive performance, nutrient digestibility, disease incidence and heat stress indices
Source: PLoS One. 2021 Jan 13;16(1):e0244922. doi: 10.1371/journal.pone.0244922 (PMC7806139; doi:10.1371/journal.pone.0244922)
Supplement: S3 File — (PDF) [file pone.0244922.s003.pdf]

Supplementary file 3. Symptoms for diagnosing the diseases of sheep reared under different farming systems

S.No.	Disease	Symptom
1	Diarrhea	Runny watery/blood stained faeces
2	Bloat	Distended abdomen, breathing difficulty, and kicking at the abdomen region
3	Pneumonia	Depression, lethargy, inappetance, sneezing, and coughing.
4	Anorexia	Lack of appetite
5	Pregnancy toxaemia	History of advanced pregnancy. Drowsy, comatose, tremors, blindness, and inappetance
6	Abscess	Swollen, pus-filled lump under the surface of the skin
7	Foot rot	Limping, lameness, raw infection of the skin between the toes. Slimy and Foul smelling toes.
8	Tick infestation	Anemia and incidence of ticks on the skin surface
