# Supplementary material for: Farming systems in sheep rearing: Impact on growth and reproductive performance, nutrient digestibility, disease incidence and heat stress indices
Source: PLoS One. 2021 Jan 13;16(1):e0244922. doi: 10.1371/journal.pone.0244922 (PMC7806139; doi:10.1371/journal.pone.0244922)
Supplement: S4 File — (PDF) [file pone.0244922.s004.pdf]

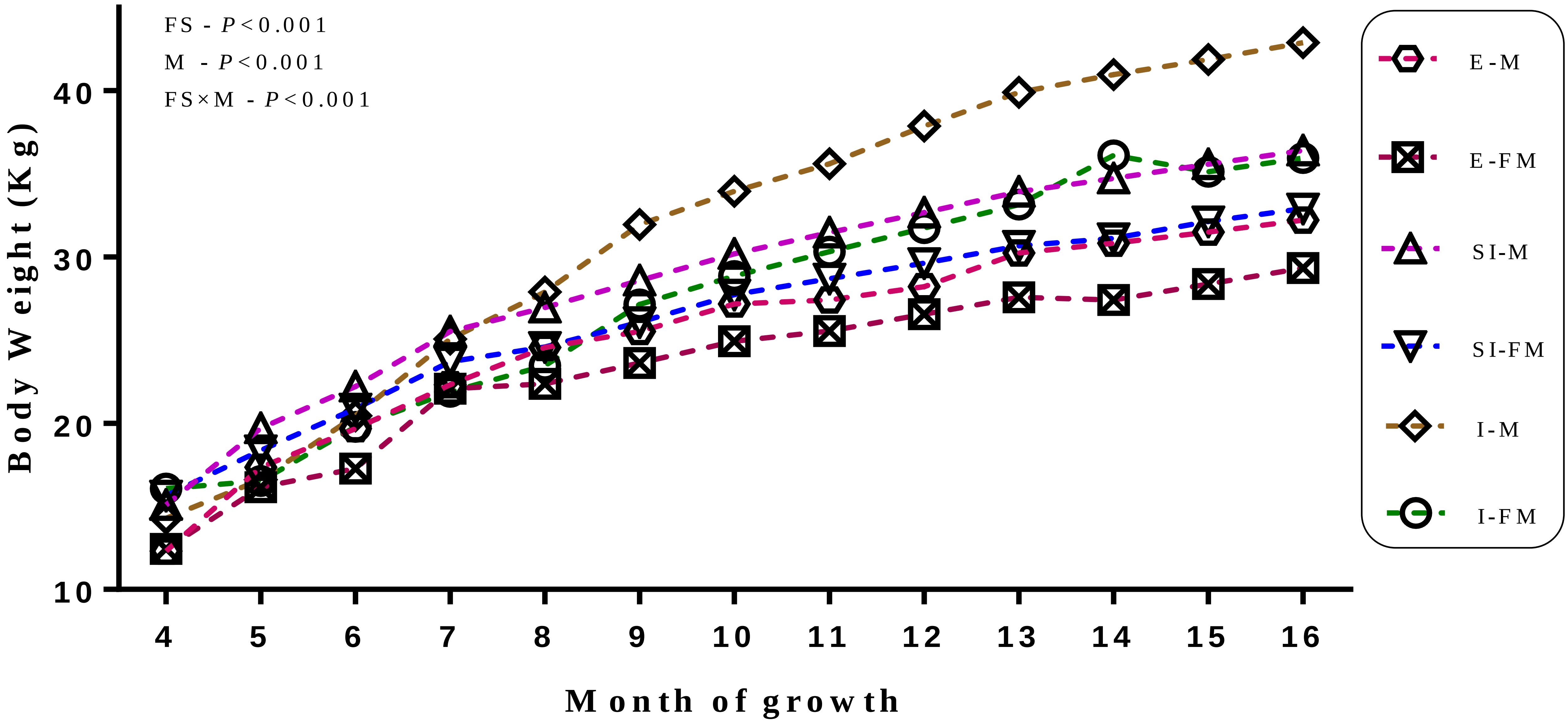

Supplementary file 4. Body weight changes with reference to the farming system

FS, Feeding system; M, Month; FS×M, Feeding system and Month interactions; E-M, Extensive-Males; E-FM, Extensive-Females; SI-M, Semi-intensive-Males; SI-FM, Semi-intensive-Females; I-M, Intensive-Males; I-FM, Intensive-Females
